# Combination of IL-6 and sIL-6R differentially regulate varying levels of RANKL-induced osteoclastogenesis through NF-κB, ERK and JNK signaling pathways

**DOI:** 10.1038/srep41411

**Published:** 2017-01-27

**Authors:** Wei Feng, Hongrui Liu, Tingting Luo, Di Liu, Juan Du, Jing Sun, Wei Wang, Xiuchun Han, Kaiyun Yang, Jie Guo, Norio Amizuka, Minqi Li

**Affiliations:** 1 Department of Bone Metabolism, School of Stomatology Shandong University, Shandong Provincial Key Laboratory of Oral Tissue Regeneration, Jinan, China; 2 Shanxi Medical University, Taiyuan, China; 3 Department of Stomatology, Qilu Hospital of Shandong University, Jinan, China; 4 Department of Developmental Biology of Hard Tissue, Graduate School of Dental Medicine, Hokkaido University, Sapporo, Japan

## Abstract

Interleukin (IL)-6 is known to indirectly enhance osteoclast formation by promoting receptor activator of nuclear factor kappa-B ligand (RANKL) production by osteoblastic/stromal cells. However, little is known about the direct effect of IL-6 on osteoclastogenesis. Here, we determined the direct effects of IL-6 and its soluble receptor (sIL-6R) on RANKL-induced osteoclast formation by osteoclast precursors *in vitro*. We found IL-6/sIL-6R significantly promoted and suppressed osteoclast differentiation induced by low- (10 ng/ml) and high-level (50 ng/ml) RANKL, respectively. Using a bone resorption pit formation assay, expression of osteoclastic marker genes and transcription factors confirmed differential regulation of RANKL-induced osteoclastogenesis by IL-6/sIL-6R. Intracellular signaling transduction analysis revealed IL-6/sIL-6R specifically upregulated and downregulated the phosphorylation of NF-κB (nuclear factor kappa-light-chain-enhancer of activated B cells), ERK (extracellular signal–regulated kinase) and JNK (c-Jun N-terminal kinase) induced by low- and high level RANKL, respectively. Taken together, our findings demonstrate that IL-6/sIL-6R differentially regulate RANKL-induced osteoclast differentiation and activity through modulation of NF-κB, ERK and JNK signaling pathways. Thus, IL-6 likely plays a dual role in osteoclastogenesis either as a pro-resorption factor or as a protector of bone, depending on the level of RANKL within the local microenvironment.

Bone remodeling is achieved by coupling between osteoblasts and osteoclasts. Osteoblasts are bone-forming cells that also support the biological function of osteoclasts, which are highly specialized multinucleated cells derived from hematopoietic precursors and are uniquely capable of lacunar bone resorption^1^,^2^. The commitment of osteoclastic precursors to mature osteoclasts requires close interaction with osteoblastic/stromal cells through cell to cell interactions and osteoclastic factors synthesized and secreted by osteoblasts, such as receptor activator of nuclear factor-κB ligand (RANKL), a key stimulant of osteoclastogenesis^3^,^4^. RANKL binds to the receptor activator of nuclear factor-κB (RANK) receptor expressed on the surface of osteoclast precursors, and subsequently activates tumor necrosis factor (TNF) receptor-associated factor-6 and downstream signaling transduction pathways, such as the mitogen-activated protein kinase (MAPK) pathway. This in turn induces activation of transcription factor nuclear factor-κB (NF-κB), leading to the formation and maturation of osteoclasts^5^,^6^. RANKL-dependent osteoclastogenesis represents the major pathway whereby osteoclastogenesis occurs, and is known as canonical osteoclast formation. However, a number of other cytokines have been shown capable of substituting RANKL to induce osteoclast formation from marrow-derived osteoclast precursors, including TNF-α, IL-1, IL-6, and IL-11^7^,^8^,^9^,^10^. These non-canonical osteoclastogenesis factors are postulated to play a critical role in pathological bone resorption, however their underlying mechanisms remain unclear.

IL-6 is a proinflammatory cytokine belonging to the gp130 family, which is also composed of IL-11, leukemia inhibitory factor (LIF), oncostatin M (OSM), cardiotrophin-1, and novel neutrophin-1/B-cell stimulatory factor-3, all of which share glycoprotein-130 (gp130) as a common signal transducer^11^. Binding of IL-6 to its receptor, IL-6R, promotes homodimerization of gp-130, thereby activating Janus kinase (JAK)/signal transducer and activator of transcription (STAT) and the MAPK pathway. IL-6 has been shown to be implicated in diseases associated with bone loss, such as postmenopausal osteoporosis^12^, rheumatoid arthritis (RA), Paget’s disease^13^, and multiple myeloma^14^, in which enhanced IL-6 levels occur. IL-6 *null* mice are protected from developing joint inflammation, and the destruction in collagen- or antigen-induced arthritis^15^,^16^, as well as the loss of bone caused by estrogen depletion, can be prevented in mice by infusion of antibody neutralizing IL-6^17^ or by IL-6 gene knockout^18^. This finding strongly suggests that IL-6 is an important mediator of pathological bone loss, and thus a potential osteoresorptive factor. Despite this, it has also been shown that IL-6 modulation of osteoclastogenesis requires assistance by soluble IL-6 receptor (sIL-6R), evidenced by the fact that IL-6 induces functional osteoclast formation only in the presence of sIL-6R^19^, indicating insufficient expression of IL-6R in osteoclastic lineage cells.

IL-6 is believed to predominantly stimulate osteoclast activity and bone resorption by indirectly inducing production of RANKL by osteoblastic/stromal cells, which in turn stimulates the commitment of osteoclast precursors into mature osteoclasts^20^. However, accumulating evidence for the direct effect of IL-6 on osteoclast activity has emerged. A RANKL-independent mechanism by which IL-6 supports human osteoclast formation has been reported by Kudo *et al*.^10^. In addition, recent studies have indicated that IL-6 directly inhibits RANKL-induced osteoclast formation^21^,^22^. Thus, the precise target cell(s) effected by IL-6 and its role in basal and inflammatory osteoclastogenesis remain controversial.

We have previously examined the impact of IL-6-deficiency on osteoclast formation *in vivo* and found that depletion of IL-6 in mice resulted in increased numbers of osteoclasts with attenuated resorptive activity, indicating separate regulation of the number and function of osteoclasts by IL-6^23^. This study therefore aimed to investigate the influence of IL-6 and sIL-6R on gradient concentrations of RANKL-induced osteogenesis and to identify the potential underlying mechanisms.

## Materials and Methods

### Cells culture and antibodies

Murine RAW264.7 monocytic cells were purchased form the Shanghai Cell Center (Shanghai, China). The α-minimum essential medium (α-MEM), penicillin/streptomycin and fetal bovine serum (FBS) were purchased from Gibco-BRL (Gaithersburg, MD, USA). Recombinant soluble mice receptor activator for nuclear factor-κB ligand (RANKL) and macrophage colony-stimulating factor (M-CSF) were from R&D Systems (Minneapolis, MN, USA). Specific antibodies against extracellular signal-regulated kinase (ERK), c-Jun N-terminal kinase (JNK), p38, RANK, nuclear factor kappa light chain enhancer of activated B cells (NF-κB), phospho-ERK (Thr^202^/Tyr^204^), phospho-JNK (Thr^183^/Tyr^185^), phospho-p38 (Thr^180^/Tyr^182^), phospho-NF-κB (Ser^536^) and horseradish peroxidase-conjugated goat anti-rabbit IgG were obtained from Cell Signaling Technology (Cambridge, MA, USA). Anti-receptor activator for nuclear factor-κB (RANK), anti-nuclear factor of activated T cells cytoplasmic 1 (NFATc1), anti-c-fos, anti-TNF receptor associated factor 6 (TRAF6), Akt, phospho-Akt (Ser^473^), and anti-β-actin antibodies were from Abcam (Cambridge, MA, USA).

### Mouse bone marrow macrophage preparation and osteoclast differentiation

All animal experiments were conducted according to the Guidelines for Animal Experimentation of Shandong University. The animal care and experimental protocol were approved by a committee of the Medical Ethics Committee for Experimental Animals, Shandong University School of Stomatology. Male, four to six-week-old C57BL/6 mice were used in this study. Primary bone marrow macrophages (BMMs) were isolated from the whole bone marrow as described previously. Briefly, mice were sacrificed by decapitation under deep anesthesia with 10% Chloral hydrate. Tibiae and femurs were isolated and flushed with α-MEM. The cells were cultured in α-MEM containing 10% FBS, 100 U/ml penicillin G, and 100 μg/ml streptomycin at 37 °C under 5% CO_2_. Non-adherent cells were layered onto a Ficoll density gradient solution and centrifuged at 440 g for 30 min at room temperature. Cells lying in the upper layer were harvested as BMMs. The cells were seeded in 6-well plate (5 × 10^5^ cells/well) or 24-well plates (3 × 10^4^ cells/well) and cultured for 6 days in α-MEM supplemented with 10% FBS, 30 ng/ml M-CSF and 10 ng/ml or 100 ng/ml RANKL in the absence or presence of 100 ng/ml IL-6 and 100 ng/ml sIL-6R. The culture medium was changed to fresh medium every other day. Similarly, the effect of IL-6 and sIL-6R on varying concentration of RANKL-induced osteoclast differentiation was also evaluated on the RAW264.7 cell line.

### Tartrate-resistant Acid Phosphatase (TRAP) Staining

TRAP staining was used to evaluate osteoclast differentiation. BMMs or RAW264.7 cells were seeded onto 24-well plates at a density of 3 × 10^4^ and cultured in α-MEM supplemented with stimulus as indicated in results section for 4 days. Cells were fixed with 4% formaldehyde for at least 15 minutes at room temperature and stained for TRAP using TRAP-staining solution: 0.1 M sodium acetate (pH 5.0) containing 0.01% naphthol AS-MX phosphate (Sigma-Aldrich) as a substrate, and 0.03% red violet LB salt (Sigma-Aldrich) as a stain for the reaction product in the presence of 50 mM sodium tartrate. Cell nucleus were counterstained with hematoxylin for 2 min. Multinucleated TRAP-positive cells with at least 3 nuclei were scored as osteoclasts.

### Resorption Pit Formation Assay

Pit formation assay was performed using the Corning Osteo Assay Surface Multiple Well Plate (Corning, Inc., Corning, NY, USA). BMMs or RAW264.7 cells were seeded onto 96-well plates at a density of 5 × 10^3^ and cultured in α-MEM supplemented with stimulus as indicated in results section for 14 days. The culture medium was replaced with fresh medium containing these reagents every 2 days. After the culture, plates were stained with Von Kossa to increase contrast between pits and surface coating and observed under a light microscope. The percentage of the resorbed areas and the number of resorption pits in three random resorption sites were measured under microscopic examination using Image-Pro Plus 6.2 software (Media Cybernetics, Silver Spring, MD, USA). The assays were performed in triplicate, and a representative view from each assay is presented.

### RNA Extraction and Real-time RT-PCR Analysis

BMMs were seeded in 24-well plates (3 × 10^4^ cells/well) and incubated with or without 100 ng/ml IL-6 and 100 ng/ml sIL-6R in the presence of 30 ng/ml M-CSF and gradient concentration of RANKL for 2 days. Total RNA was isolated from osteoclasts using an RNeasy Mini kit (Qiagen, Valencia, CA, USA), quantitative real-time PCR analysis was performed to test mRNA expression of TRAP, Cathepsin K (CK), Calcitonin receptor (CTR) and matrix metalloproteinase (MMP)-9 using the primer sequences shown in Table 1. All quantitative reverse transcription-PCRs were performed using Roche LightCycler 480 Real-Time PCR system (Roche, Sussex, UK), and all samples were run in triplicate. The cycling conditions were as follows: 40 cycles of 95 °C for 3 s and 60 °C for 30 s. Relative quantities of the tested genes were normalized to GAPDH mRNA. Analysis of the relative quantitation required calculations based on the threshold cycle, i.e. the cycle number at which the amplification plot crosses a fixed threshold above the baseline (*C*t). Relative quantitation was performed using the comparative ΔΔ*C*t method according to the manufacturer’s instructions.

### Co-immunoprecipitation assay

BMMs were plated in 10 cm-diameter dishes at a density of 2 × 10^5^ cells/well and cultured with 30 ng/ml M-CSF. When the cells grew to confluent, they were pre-treated with or without 100 ng/ml IL-6 and 100 ng/ml sIL-6R for 4 h. The cells were then stimulated with low (10 ng/ml) or high (50 ng/ml) concentration of RANKL for 0, 5, 15 or 30 min and then subjected to co-immunoprecipitation assay using Immunoprecipitation (IP) Kit (Proteintech Group, Chicago, USA) according to the manufacturer’s instructions. Briefly, cells were washed three times with ice-cold phosphate-buffered saline and lysed in IP lysis buffer. Cell debris was removed by centrifugation at 10,000 rpm for 15 min at 4 °C followed by protein concentration measurement using the BCA method. After that, cell lysates were incubated with anti-RANK antibody at 4 °C for overnight and rotated with protein A sepharose beads slurry at 4 °C for 4 h. The beads were washed three times with washing buffer. The immunoprecipitates or whole-cell lysates were subjected to sodium dodecyl sulfate-polyacrylamide gel electrophoresis (SDS-PAGE) and transferred to Immobilon polyvinylidene difluoride membranes (Millopore Corporation, Billerica, MA, USA). The membranes were immunoblotted with rabbit anti-TRAF6 for 1 h at room temperature followed by horseradish peroxidase-conjugated goat anti-rabbit IgG and visualized by the ECL system (SmartChemi 420; Sagecreation, Beijing, China).

### Western Blot Analysis

BMMs were seeded in six-well plates at a density of 1 × 10^5^ cells/well and cultured with low (10 ng/ml) or high (50 ng/ml) level of RANKL and 30 ng/ml M-CSF in the presence or absence of 100 ng/ml IL-6/sIL-6R. After 2 days, the total protein was collected for NFATc1 and c-fos immunoblotting. To detect the effect of IL-6/sIL-6R on RANKL-activated intracellular signal transduction cascades, BMMs were seeded in six-well plates at a density of 1 × 10^5^ cells/well. When the cells were confluent, they were pre-treated with or without 100 ng/ml IL-6 and 100 ng/ml sIL-6R for 4 h. The cells were then stimulated with low (10 ng/ml) or high (50 ng/ml) concentration of RANKL for 0, 15, 30 or 60 min. After that, cell lysates were prepared with RIPA lysis buffer (Beyotime, Beijing, China). Cell debris was removed by centrifugation at 10,000 rpm for 15 min at 4 °C followed by protein concentration measurement using the BCA method. Protein was denatured by boiling for 5 min before electrophoresis. Each protein sample was subjected to 6% SDS–PAGE, and transferred to Immobilon polyvinylidene difluoride membranes. The membranes were blocked with 5% BSA in TBS-T for 1 h, and incubated with rabbit anti-NFATc1, anti-c-fos, anti-phospho-p38, anti-p38, anti-phospho-ERK, anti-ERK, anti-phospho-JNK, anti-JNK, anti-phospho-Akt, anti-Akt, anti-phospho-NF-κB and anti-NF-κB antibodies for 1 h at room temperature. The membranes were then washed three times with TBS-T for 5 min each and incubated with horseradish peroxidase-conjugated goat anti-rabbit IgG at a dilution of 1:1000. After three washes with TBS-T, the immunoreactive bands were visualized with ECL detection system. β-actin serves as loading control.

### Statistics

The data were expressed as means ± standard deviations (SD). All experiments were performed in triplicate. SPSS 16.0 software was used to analysis the obtained data. One-way ANOVA was used for multiple groups’ comparison, and the mean value of each group was compared using the Student-Newman-Keuls (SNK) test. Results were Student’s t-test was used for statistical analysis. *P* < 0.05 was considered statistically significant.

## Results

### IL-6 induces osteoclast formation by murine BMMs in the presence of sIL-6R

**Figure 1:**
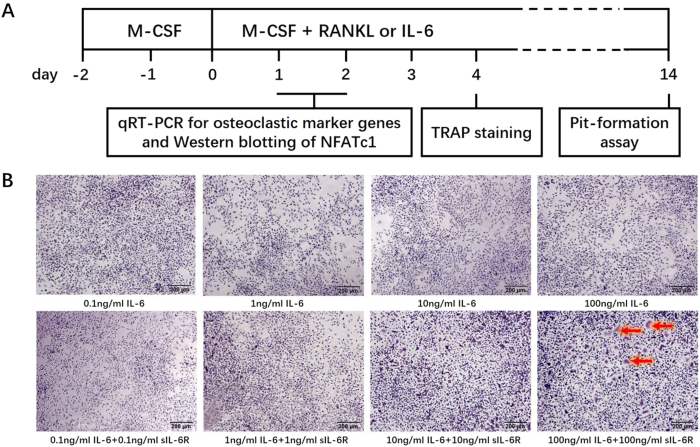
IL-6 slightly induces osteoclast-like cells formation by mice BMMs only in the presence of sIL-6R. (**A**) Schematic diagram of the culture system and assays used in this *in vitro* study. (**B**) BMMs were treated with various concentration of IL-6 alone or IL-6 and sIL-6R in combination in the presence of M-CSF (30 ng/ml) for 5 days. Then, cells were fixed with 4% PFA and subjected to TRAP staining. TRAP-positive multinucleated cells (more than three nuclei) were counted under a light microscope (original magnification, ×100). Red arrows indicate the induced TRAP-positive multinucleated cells.

The culture system and assays are schematically presented in Fig. 1A. To investigate whether sIL-6R is indispensable for IL-6 induction of osteoclast formation, BMMs were cultured with IL-6 and sIL-6R separately or in combination in the presence of 30 ng/ml M-CSF for 4 days. TRAP staining was then performed. RANKL was used as a positive control stimulator of osteoclast formation. As shown in Fig. 1B, neither IL-6 (0.1–100 ng/ml) nor sIL-6R (0.1–100 ng/ml) alone stimulated TRAP-positive multinucleated osteoclasts. However, when IL-6 and sIL-6R were simultaneously applied to the BMM cultures, a small but significant increase in osteoclast formation was observed at concentrations of 100 ng/ml. These results suggest that the biological expression of membrane-bound IL-6R (mIL-6R) in BMMs is not sufficient for IL-6 to promote osteogenesis and exogenous sIL-6R supplementation is required to induce significant osteoclast formation by IL-6.

### IL-6 suppresses high concentration RANKL-induced osteoclast formation whereas sIL-6R has no effect on RANKL-induced osteoclastogenesis

**Figure 2:**
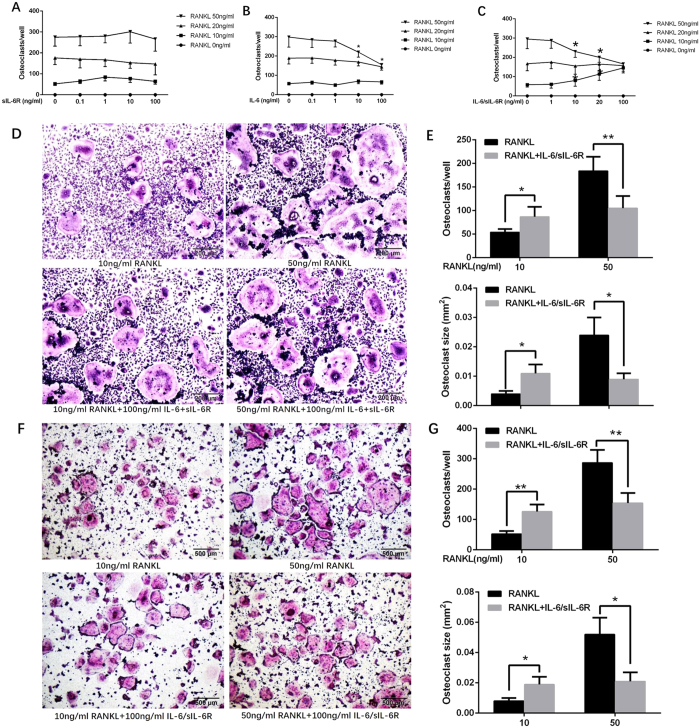
IL-6/sIL-6R differentially regulate osteoclast-like cells formation from osteoclast precursors induced by varying concentration of RANKL. BMMs were cultured in the presence of series concentration of RANKL (0–50 ng/ml) and M-CSF (30 ng/ml) with or without gradient concentration (0–100 ng/ml) of sIL-6R (**A**), IL-6 (**B**) alone or IL-6 plus sIL-6R (**C**). After 4 days’ culture, TRAP staining was performed to visualize mature osteoclasts followed by cell count. (**D**) Representative images of TRAP staining for BMMs cultured with M-CSF (30 ng/ml) and low level of RANKL (10 ng/ml) or high level of RANKL (50 ng/ml) in the presence or absence of IL-6/sIL-6R (100 ng/ml) (original magnification, ×100). (**E**) The number and mean size of TRAP-positive multinucleated cells for panels (D). (**F**) Representative images of TRAP staining for RAW264.7 cells cultured with M-CSF (30 ng/ml) and low level of RANKL (10 ng/ml) or high level of RANKL (50 ng/ml) in the presence or absence of IL-6/sIL-6R (100 ng/ml) (original magnification, ×40). (**G**) The number and mean size of TRAP-positive multinucleated cells for panels (**F**). All experiments were carried out at least 3 times, and data are expressed as mean ± SD, (*p < 0.05, **p < 0.01).

To determine the effect of IL-6 or sIL-6R alone on RANKL-induced osteoclast formation, murine BMMs were cultured in the presence of a series of RANKL concentrations, with or without gradient concentrations of IL-6 and sIL-6R alone or in combination. After 4 days in culture, TRAP staining was performed to visualize mature osteoclasts. As shown in Fig. 2A–C, 10 ng/ml RANKL yielded a few multinucleated osteoclast-like cells, and both the number and size of the osteoclast-like cells increased with increased concentration of RANKL. Exposure to sIL-6R did not affect the number of osteoclast-like cells induced by different concentrations of RANKL (Fig. 2A). However, high dose IL-6 (>10 ng/ml) suppressed 50 ng/ml RANKL-induced osteoclastogenesis but had little effect on lower concentrations of RANKL-induced osteoclast formation (≤20 ng/ml) (Fig. 2B). These results suggest that IL-6 rather than sIL-6R exerts a negative regulatory effect on RANKL-induced osteoclast formation and that this effect is RANKL concentration-dependent.

### Combined IL-6 and sIL-6R differentially regulate RANKL-induced osteoclastogenesis

To evaluate the direct effect of combined application of IL-6 and sIL-6R on RANKL-induced osteoclast formation, murine BMMs cells were cultured in the presence of a concentration series of RANKL with or without 100 ng/ml IL-6/sIL-6R. After 4 days in culture in the presence of RANKL, a few multinucleated osteoclast-like cells were observed in 10 ng/ml RANKL-treated BMMs, and both the number and size of the osteoclast-like cells increased with increased concentration of RANKL. Characteristic osteoclast-like cells with TRAP-positive large cell bodies and numerous nuclei were notably identified in the BMMs exposed to 50 ng/ml RANKL (Fig. 2C and D). An increased RANKL concentration to 100 ng/ml failed to yield more osteoclast-like cells, indicating that 50 ng/ml is the saturated RANKL concentration for induction of osteoclastogenesis by BMMs (data not shown). Strikingly, although IL-6 or sIL-6R alone had no effect on osteoclast differentiation of BMMs, addition of 100 ng/ml IL-6/sIL-6R strongly favored RANKL-induced osteoclast formation at the concentration of 10 ng/ml RANKL, as evaluated by the number of TRAP-positive multinuclear cells (Fig. 2C–E). However, when the RANKL concentration was increased to 50 ng/ml, exogenous application of IL-6/sIL-6R highly suppressed the formation of TRAP-positive multinuclear cells compared with the control (Fig. 2C–E). A similar change pattern in osteoclastogenesis was found in RAW264.7 cells (Fig. 2F and G). These results indicate that IL-6/sIL-6R mediation of RANKL-induced osteoclastogenesis is RANKL concentration-dependent. In short, IL-6/sIL-6R positively and negatively regulated low- and high-concentration RANKL-induced osteoclast differentiation, respectively.

### Combined IL-6 and sIL-6R differentially regulate the resorptive potential of RANKL-induced osteoclasts

**Figure 3:**
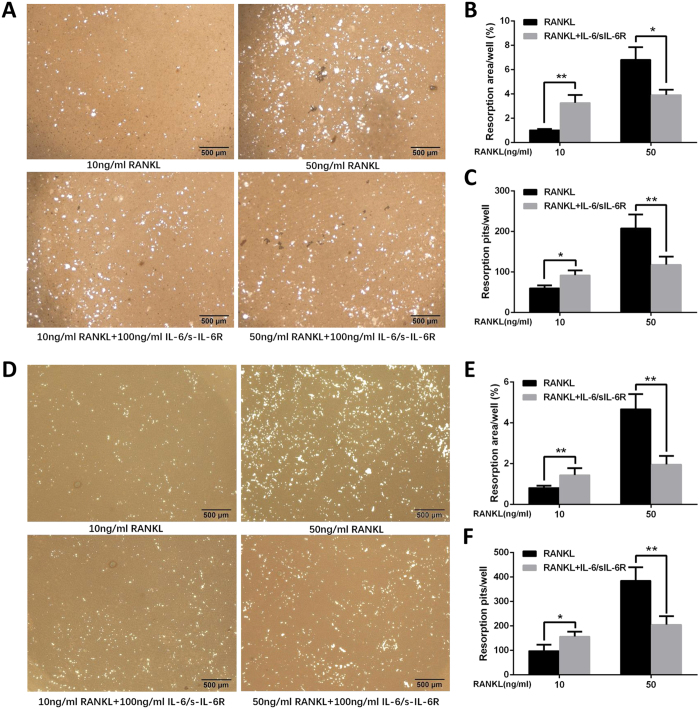
IL-6/sIL-6R differentially regulate resorption ability of varying concentration of RANKL-induced osteoclast. (**A**) RAW264.7 cells were cultured with low level (10 ng/ml) or high level (50 ng/ml) of RANKL and M-CSF (30 ng/ml) in the presence or absence of IL-6/sIL-6R (100 ng/ml). After 14 days, cells were removed and the mineral-coated wells were counterstained by Von Kossa. Resorption pits were examined by light microscope. (**B**) The percentage of mineral-coated surface occupied by resorption lacunae from panels (**A**). (**C**) The quantification of the number of resorption pits from panels (**A**). (**D**) BMMs were cultured with low level (10 ng/ml) or high level (50 ng/ml) of RANKL and M-CSF (30 ng/ml) in the presence of IL-6/sIL-6R (100 ng/ml). After 14 days, cells were removed and the mineral-coated wells were counterstained by Von Kossa. Resorption pits were examined by light microscope. (**E**) The percentage of mineral-coated surface occupied by resorption lacunae from panels (**D**). (**F**) The quantification of the number of resorption pits from panels (**D**). Data are expressed as mean ± SD, (*p < 0.05, **p < 0.01).

Mature osteoclasts are capable of degrading bone matrix and forming bone resorption pits in bovine cortical bone and dentin slices. To determine the effect of IL-6/sIL-6R on osteoclastic activity of varying concentrations of RANKL-induced osteoclasts, we employed a pit formation assay. Resorption pits were observed on mineral-coated plastic dish when the BMMs were cultured with 50 ng/ml RANKL (Fig. 3A–C). However, few resorption pits were generated by the BMMs cultured with 10 ng/ml RANKL (Fig. 3A–C). When IL-6/sIL-6R was added, the number and area of resorption pits were remarkably enlarged for 10 ng/ml RANKL-treated BMMs. In contrast, the addition of IL-6/sIL-6R resulted in a significant decrease in the number and area of resorption pits induced by 50 ng/ml RANKL-treated BMMs (Fig. 3A–C). A similar pattern in pit formation was found using RAW264.7 cells (Fig. 3D–F). These results imply that IL-6/sIL-6R potentiates the osteoclastic ability of osteoclasts induced by low-concentration RANKL but compromise that of osteoclasts induced by high-concentration RANKL. In the subsequent experiments, 10 ng/ml and 50 ng/ml concentrations are designated as low- and high level RANKL, respectively, and were used to investigate the underlying mechanisms of differential regulation of RANKL-induced osteoclast formation by IL-6/sIL-6R.

### Combined IL-6 and sIL-6R differentially regulate RANKL-induced expression of osteoclast-specific genes and transcriptional factors

**Figure 4:**
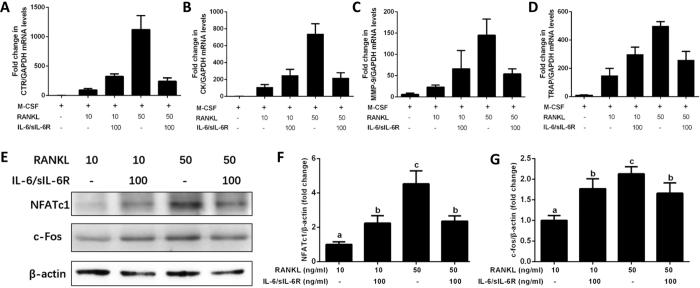
IL-6/sIL-6R differentially regulate varying concentration of RANKL-induced expression of osteoclast-specific genes and transcriptional factors by osteoclast precursors. BMMs cells were cultured with low level (10 ng/ml) or high level (50 ng/ml) of RANKL and M-CSF (30 ng/ml) in the presence or absence of IL-6/sIL-6R (100 ng/ml) for 2 days. After that, total RNA was extracted and subjected to quantitative real-time PCRs using probes specific for *CTR* (**A**), *cathepsin K* (**B**), *MMP*-*9* (**C**), *TRAP* (**D**) and *GAPDH*. The levels of osteoclast specific genes mRNA expression were normalized to *GAPDH* expression. (**E**) The same protein amounts of cell lysates were subjected to SDS-PAGE, followed by western blotting with antibodies specific to NFATc1and c-fos. β-actin was used as a loading control. Histograms at the right side of the Western blot showed the bands’ intensities for NFATc1 (**F**) and c-fos (**G**) quantified by Image-J software (National Institutes of Health, Bethesda, MD, USA) and represented as a ratio to actin signals. The data are representative of three independent experiments expressed as means ± SD. Different letters indicate significant differences between groups (*p* < 0.05).

We next confirmed the differential regulation of IL-6/sIL-6R on varying concentrations of RANKL-induced osteoclast differentiation by analyzing the expression levels of osteoclastic marker genes and critical transcriptional factors. BMMs were cultured in low or high RANKL concentrations and 30 ng/ml M-CSF in the presence or absence of 100 ng/ml IL-6/sIL-6R. After 2 days of osteoclastogenic induction, expression levels of TRAP, CTR, cathepsin K and MMP-9 mRNA were increased in the presence of RANKL in a dose-dependent manner, under the saturated concentration of 50 ng/ml (Fig. 4A–D). Furthermore, IL-6/sIL-6R strongly induced and suppressed expression of all these marker genes in low- and high-level RANKL, respectively, as compared with their respective controls (Fig. 4A–D). Western blotting showed that expression levels of both NFATc1 and c-fos induced by low- and high-level RANKL, were upregulated and suppressed in response to IL-6/sIL-6R, respectively (Fig. 4E–G). Taken together, these results clearly demonstrated that IL-6/sIL-6R acted on BMMs to differentially regulate RANKL-induced expression of osteoclast-specific genes and transcription factors, leading to differential modulation of the subsequent osteoclast differentiation.

### NF-κB, ERK and JNK MAPKs pathways are specifically involved in IL-6/sIL-6R-mediated differential regulation of osteoclastogenesis by varying RANKL concentrations

**Figure 5:**
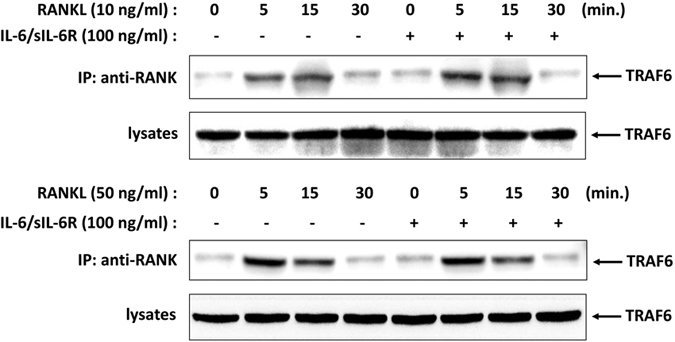
RANK-TRAF6 interaction in BMMs is not altered by IL-6/sIL-6R stimulation. BMMs cells were pretreated with or without IL-6/sIL-6R (100 ng/ml) for 4 h followed by low level (10 ng/ml) or high level (50 ng/ml) of RANKL treatment for the indicated times. Cell lysates were immunoprecipitated with anti-RANK antibody and coprecipitated TRAF6 was detected by immunoblotting with anti-TRAF6 antibody. Total cell lysate content of TRAF6 were determined by immunoblot.

To gain insight into the mechanism of differential mediation of RANKL-induced osteoclastogenesis by IL-6/sIL-6R, we performed immunoblot analysis of molecules known to be critically involved in RANKL signaling in RANKL-induced BMMs pretreated with or without exogenous IL-6 and sIL-6R. RANKL-RANK interaction recruits TRAF6 and activates downstream signaling, including MAPKs, Akt and NF-κB pathways. We therefore examined whether RANK-TRAF6 interaction was altered by IL-6/sIL-6R. As shown in Fig. 5, association of TRAF6 with RANK was observed to peak 5 minutes after both low- and high-level RANKL treatment and declined thereafter. Pretreatment of IL-6/sIL-6R did not affect the association between TRAF6 and RANK. These results demonstrate that IL-6/sIL-6R exerted no effect on the formation of the RANK-TRAF6 complex in response to RANKL stimulation in BMMs.

**Figure 6:**
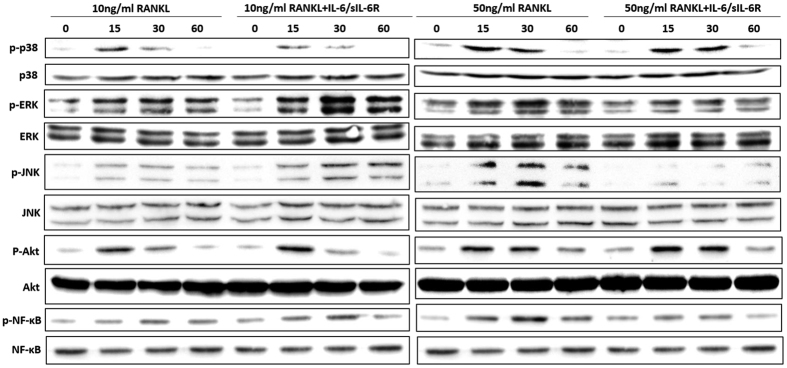
The effect of IL-6/sIL-6R on the activation of MAPKs, Akt and NF-κB signaling pathway in BMMs. BMMs cells were pretreated with or without IL-6/sIL-6R (100 ng/ml) for 4 h followed by low level (10 ng/ml) or high level (50 ng/ml) of RANKL treatment. Cell lysates were collected at the indicated time points and subjected to Western blot analysis with specific antibodies against p-38, phosphor-p-38, ERK, phosphor-ERK, JNK, phosphor-JNK, Akt, phosphor-Akt, NF-κB, phosphor-NF-κB to determine the level of phosphorylation of indicated signaling molecules. ERK served as a loading control. The bands’ intensities were quantified by Image-J software and represented as a ratio to ERK signals.

Next, the effects of IL-6/sIL-6R on TRAF6 downstream signaling pathways, including NF-κB, MAPKs (ERK, JNK and p38) and Akt, were analyzed. As evidenced by the immunoblot assay in Fig. 6, RANKL induced NF-κB phosphorylation, but this was significant only at high level concentration. When BMMs were pretreated with IL-6/sIL-6R, NF-κB phosphorylation induced by low- and high-level RANKL was strongly potentiated and suppressed to a level identical to that of BMMs cultured with 10 ng/ml RANKL and IL-6/sIL-6R, respectively. With respect to MAPK signaling pathways, IL-6/sIL-6R pretreatment enhanced and attenuated ERK and JNK activation induced by low- and high-level RANKL, respectively, exhibiting a change pattern similar to that of NF-κB. However, activation of p38 and Akt were not affected by IL-6/sIL-6R treatment. These results indicate that the NF-κB, ERK and JNK signaling pathways are implicated in the mechanism by which IL-6/sIL-6R regulates different concentrations of RANKL-induced osteoclastogenesis.

## Discussion

This study implicates IL-6/sIL-6R as a critical modulator for the control of osteoclastogenesis. IL-6 together with its soluble receptor, sIL-6R, promoted bone resorption in the context of low RANKL level while inhibited it when RANKL level increased. Our findings highlight the unique role of IL-6 in direct mediation of bone resorption other than its well-known pro-inflammatory function and suggest that activation of IL-6R is essential and a clinically relevant factor for the maintenance of bone erosion homeostasis.

Several studies indicate an indirect promotive effect of IL-6 on osteoclast formation and activation through inducing RANKL production in osteoclast-supporting cells^24^,^25^,^26^. Meanwhile, IL-6 synthesized by local osteoblasts and monocytes may act on osteoclast precursors to influence osteoclastogenesis^27^,^28^. However, recent studies suggest a negative rather than positive effect of IL-6 on osteoclast differentiation, through osteoclastogenesis inhibition^21^,^22^. Our data support this concept because IL-6 attenuated high level RANKL-induced osteoclast formation and bone resorptive activity regardless of whether sIL-6R was present. Conversely, the combination of IL-6 and sIL-6R potentiated rather than inhibited low level RANKL-induced osteoclastogenesis and osteoclastic function. This result was unexpected and indicative of the existence of a bidirectional regulatory mechanism for RANKL-induced osteoclastogenesis by IL-6 in the presence of sIL-6R. Furthermore, NFATc1 and c-fos, two of the most important transcription factors for osteoclast commitment, displayed the same trend change, implying that IL-6/sIL-6 differentially mediates varied concentrations of RANKL-induced osteoclastogenesis at transcriptional level.

During osteoclast development, RANKL binds to RANK receptor expressed in osteoclast precursor cells and recruits TRAF6 to specific domains within the cytoplasmic domain of RANK^29^, which is essential for activation of downstream signaling cascades. Thus, we evaluated the effect of IL-6/sIL-6R on RANKL-triggered RANK-TRAF6 interaction in murine BMMs and found that IL-6/sIL-6R did not change the association of RANK and TRAF6 induced by either low- or high level RANKL. These results indicate that IL-6/sIL-6R inhibition of osteoclastogenesis is not achieved by interfering with the RANKL-induced RANK-TRAF6 interaction.

NF-κB signaling is among a cascade of intracellular signal transduction factors activated by RANKL-RANK interaction and plays a centrally important role in osteoclast differentiation. The classical NF-κB signaling pathway involves activation of the IKK complex, which phosphorylates IκBα and targets it for ubiquitin-dependent degradation, therefore leading to translocation of NF-κB to the nucleus^30^. In the alternative IκB-independent pathway, direct phosphorylation of NF-κB subunit p65 by IKK also modulates NF-κB transcription activity^31^. Our results showed that NF-κB subunit p65 phosphorylation, induced by low- and high-level RANKL, was strongly potentiated and suppressed by IL-6/sIL-6R, respectively. This finding strongly supports involvement of the NF-κB signaling pathway in IL-6/sIL-6R-mediated differential regulation of varying concentrations of RANKL-induced osteoclast differentiation.

MAPKs (ERK, JNK, and p38) and Akt signaling pathways are known to be implicated in RANK signaling transduction and are required for subsequent osteoclast formation^32^,^33^,^34^,^35^. In the present study, we demonstrated that IL-6/sIL-6R specifically upregulated and attenuated ERK and JNK activation at low- and high-level RANKL, respectively. This suggests that IL-6/sIL-6R-mediated differential regulation of RANKL-induced osteoclast differentiation might be achieved through mediation of ERK and JNK signaling pathways. However, the activation of p38 and Akt were not changed in response to IL-6/sIL-6R. This may be due to cross-talk between RANKL-RANK-activated signaling pathways and IL-6-sIL-6R-gp130-activated signaling pathways, whereby ERK and JNK are shared by both signaling cascades but p38 and Akt are not.

IL-6 exerts its biological roles by activating two main signal transduction pathways: SHP-2/ERK and JAK/STAT^36^. In the present study, interplay between IL-6-IL-6R-gp130 trans-signaling with RANKL-RANK signaling transduction was not investigated. However, recent research has shown that IL-6-mediated inhibition of RANKL-induced osteoclast formation is achieved by interfering with the commitment of osteoclast progenitors to mature osteoclasts. In this process, serine727 phosphorylation of STAT-3 by IL-6-IL-6R plays a crucial role^22^. Because RANKL-RANK interaction activates a variety of downstream signaling pathways to enable osteoclastic differentiation, it appears that cross-talk between IL-6 trans-signaling and the RANKL-RANK signaling pathway inhibited high level RANKL-induced osteoclastogenesis.

In the current study, IL-6 induction of osteoclast differentiation was only observed in the presence of sIL-6R, implying that the unsaturated expression of mIL-6R on osteoclast precursors and the full realization of IL-6 biological function requires the aid of sIL-6R. More importantly, we found that IL-6 alone did not affect low level RANKL-induced osteoclast differentiation, but the combination of IL-6 and sIL-6R potentiated it. This distinct effect is likely due to the complementary effect of sIL-6R, which can substitute IL-6R to bind with IL-6 to form a complex with gp130. This in turn initiates IL-6-induced receptor signaling^37^, thus augmenting the IL-6 trans-signaling system to a level sufficient to exert a significant impact on the commitment of osteoclast precursors to mature osteoclast by RANKL. A similar synergistic effect has also been found for TNF-α and IL-1-mediated osteoclastogenesis, in which these two cytokines induce osteoclast formation only in the presence of permissive levels of RANKL that are not sufficient to stimulate osteoclastogenesis^38^,^39^.

The RANKL-dependent regulation of osteoclast formation by IL-6/sIL-6R might reflect pathological processes of the local microenvironment in autoimmune diseases associated with bone destruction, such as RA, and whether this effect is stimulatory or inhibitory may be dependent on disease stage. Based on evidence in this study, we postulate that in the initial stage of disease, when RANKL levels in the local microenvironment are relatively low, the high levels of IL-6 and sIL-6R secreted by active immune cells increase osteoclast differentiation and function in two ways: by (1) indirectly acting on stromal cells or osteoblasts to stimulate RANKL production; (2) directly promoting the commitment of osteoclast precursors to mature osteoclasts. Both ways activate osteoclastic bone resorption and contribute to bone and articular cartilage erosion. However, in the progressive or late stage of disease, when RANKL level is high, IL-6 and sIL-6R may serve to balance the high RANKL concentration produced in the bone microenvironment and suppress RANKL-induced osteoclast formation, thus protecting the bone against over-resorption. In this case, IL-6/sIL-6R reflects a protective mechanism on the skeleton.

**Figure 7:**
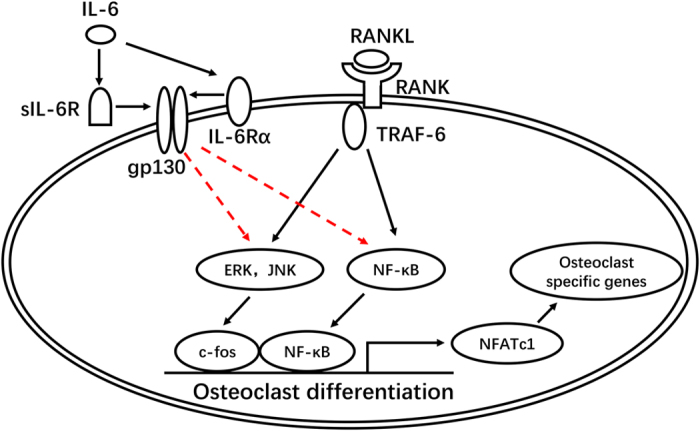
Schematic diagram of IL-6/sIL-6R differential regulation of varying concentration of RANKL-induced osteoclast differentiation and activity by osteoclast precursors. The RANKL-RANK interaction activates downstream signaling pathways such as NF-κB, MAPKs (p38, ERK and JNK) and Akt, which subsequently initiate the expression of osteoclastic transcriptional factors including NFATc1 and c-fos and osteoclast specific genes, thus leading to the commitment to mature osteoclast with potent bone resorptive activity. IL-6/sIL-6R differentially regulate RANKL-induced osteoclast formation via specifically modulating phosphorylation of NF-κB, ERK and JNK in a RANKL concentration-dependent manner, i.e., a stimulatory effect in the condition of low level of RANKL while an inhibitory effect when the level of RANKL remarkably enhanced.

In conclusion, we have provided *in vitro* evidence of differential regulation of the IL-6/sIL-6R system on osteoclast differentiation in murine osteoclast precursors and have shown that the stimulatory or inhibitory effect is dependent on the level of RANKL. Furthermore, we have demonstrated that this effect was achieved predominantly through modulation of NF-κB and ERK/JNK MAPK signaling pathways, as well as downstream transcription factors including NFATc1 and c-fos (schematically summarized in Fig. 7). These findings provide a rationale for the critical role of IL-6 in inflammatory and metabolic osteolytic diseases, such as RA, and in postmenopausal osteoporosis.

## Additional Information

**How to cite this article**: Feng, W. *et al*. Combination of IL-6 and sIL-6R differentially regulate varying levels of RANKL-induced osteoclastogenesis through NF-κB, ERK and JNK signaling pathways. *Sci. Rep.* **7**, 41411; doi: 10.1038/srep41411 (2017).

**Publisher's note:** Springer Nature remains neutral with regard to jurisdictional claims in published maps and institutional affiliations.

## Figures and Tables

**Table 1:** Oligonucleotide primers used for real-time PCR.

Gene name		Oligonucleotide Sequence (5′ → 3′)
TRAP	Forward	ACACAGTGATGCTGTGTGGCAACTC
	Reverse	CCAGAGGCTTCCACATATATGATGG
Cathepsin K	Forward	AGGCGGCTATATGACCACTG
	Reverse	CCGAGCCAAGAGAGCATATC
Calcitonin receptor	Forward	ACCGACGAGCAACGCCTACGC
	Reverse	GCCTTCACAGCCTTCAGGTAC
MMP-9	Forward	CGTCGTGATCCCCACTTACT
	Reverse	AGAGTACTGCTTGCCCAGGA
GAPDH	Forward	TGGCCTTCCGTGTTCCTAC
	Reverse	GAGTTGCTGTTGAAGTCGCA
