# Author Correction: Combination of IL-6 and sIL-6R differentially regulate varying levels of RANKL-induced osteoclastogenesis through NF-κB, ERK and JNK signaling pathways

**DOI:** 10.1038/s41598-022-07357-3

**Published:** 2022-03-02

**Authors:** Wei Feng, Hongrui Liu, Tingting Luo, Di Liu, Juan Du, Jing Sun, Wei Wang, Xiuchun Han, Kaiyun Yang, Jie Guo, Norio Amizuka, Minqi Li

**Affiliations:** 1 Department of Bone Metabolism, School of Stomatology Shandong University, Shandong Provincial Key Laboratory of Oral Tissue Regeneration, Jinan, China; 2 Shanxi Medical University, Taiyuan, China; 3 Department of Stomatology, Qilu Hospital of Shandong University, Jinan, China; 4 Department of Developmental Biology of Hard Tissue, Graduate School of Dental Medicine, Hokkaido University, Sapporo, Japan

Correction to: *Scientific Reports* 10.1038/srep41411, published online 27 January 2017

This Article contains errors.

As a result of errors in figure assembly, incorrect representative images were used in several figures. In particular, in Figure 2D, the image for 10 ng/mL RANKL + 100 ng/mL IL-6/sIL-6R, was incorrectly derived from the same image as 50 ng/mL RANKL + 100 ng/mL IL-6/sIL-6R. The corrected Figure 2D is shown below.

**Figure 2D:**
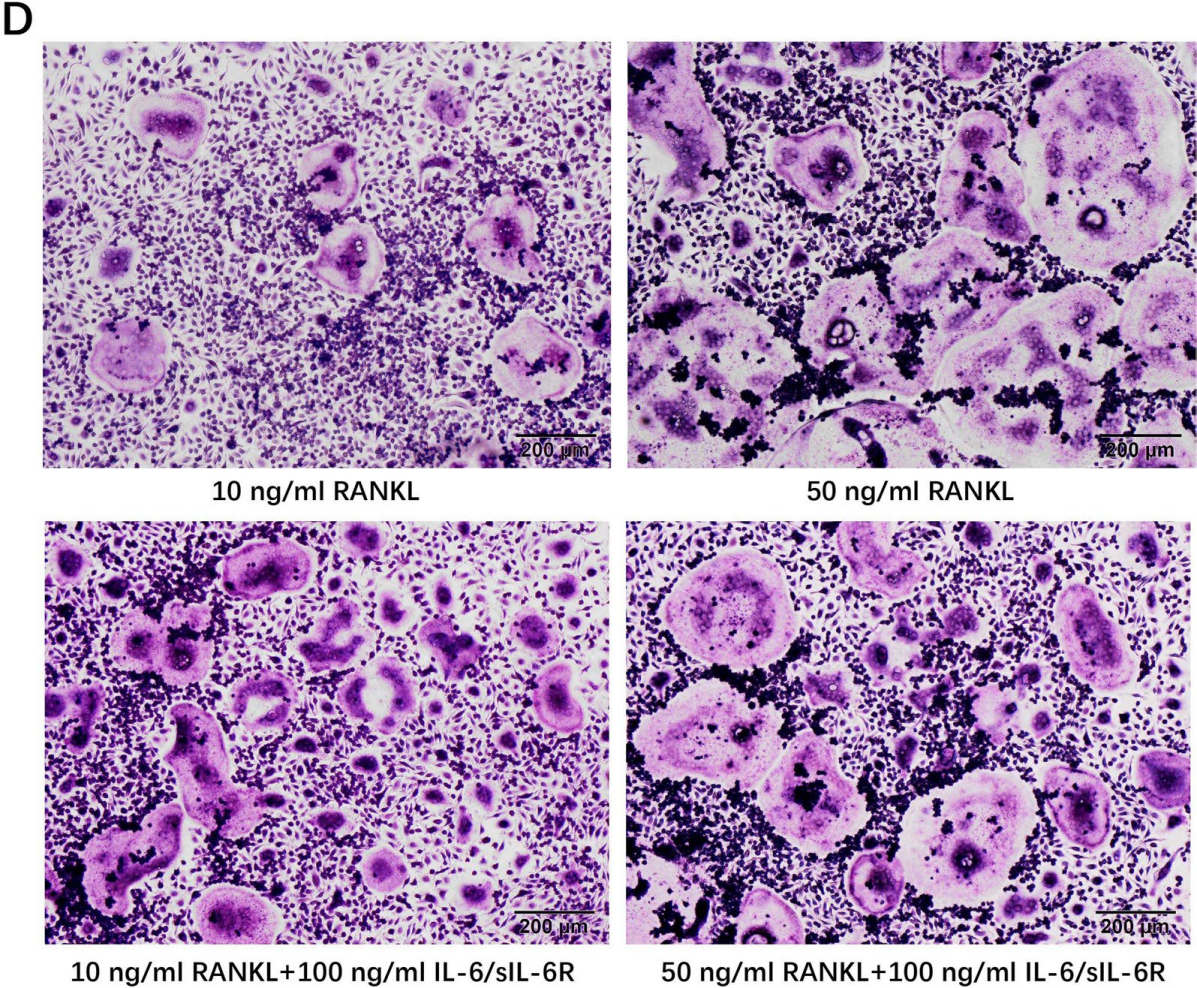
Representative images of TRAP staining for BMMs cultured with M-CSF (30 ng/ml) and low level of RANKL (10 ng/ml) or high level of RANKL (50 ng/ml) in the presence or absence of IL-6/sIL-6R (100 ng/ml) (original magnification, × 100).

In Figure 3D, the image for 10 ng/mL RANKL was incorrectly derived from the same image as 10 ng/mL RANKL + 100 ng/mL IL-6/sIL-6. The correct Figure 3D is shown below.

**Figure 3D:**
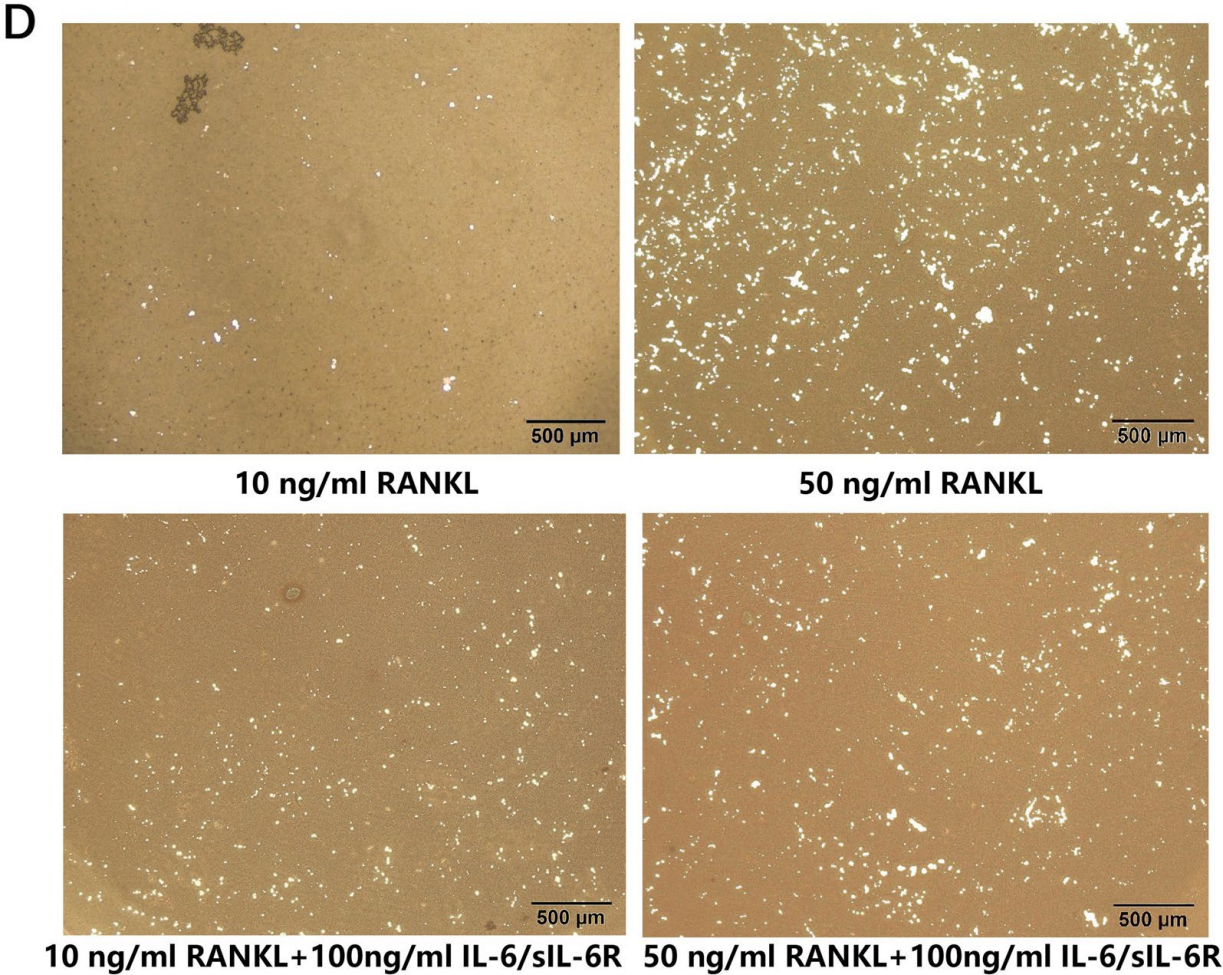
BMMs were cultured with low level (10 ng/ml) or high level (50 ng/ml) of RANKL and M-CSF (30 ng/ml) in the presence of IL-6/sIL-6R (100 ng/ml). After 14 days, cells were removed and the mineral-coated wells were counterstained by Von Kossa. Resorption pits were examined by light microscope.

Finally, in Figure 6 the image for 10 ng/mL RANKL NF-κB is a duplicate of 50 ng/mL RANKL NF-κB. The corrected Figure 6 is shown below.

**Figure 6:**
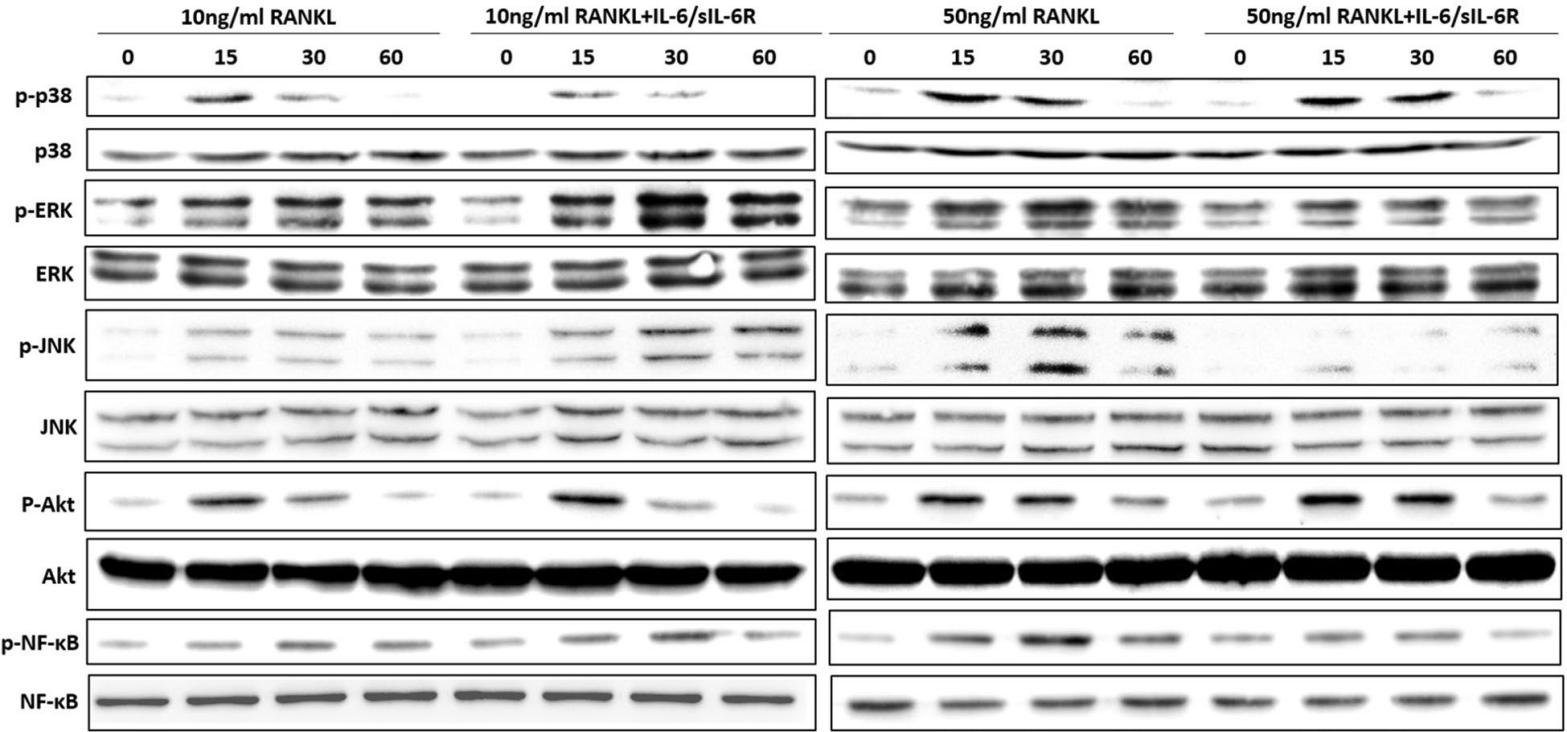
BMMs cells were pretreated with or without IL-6/sIL-6R (100 ng/ml) for 4 h followed by low level (10 ng/ml) or high level (50 ng/ml) of RANKL treatment. Cell lysates were collected at the indicated time points and subjected to Western blot analysis with specific antibodies against p-38, phosphor-p-38, ERK, phosphor-ERK, JNK, phosphor-JNK, Akt, phosphor-Akt, NF-κB, phosphor-NF-κB to determine the level of phosphorylation of indicated signaling molecules. ERK served as a loading control. The bands’ intensities were quantified by Image-J software and represented as a ratio to ERK signals.

These changes do not affect the conclusions of the Article.

